# Implications of *CD36* Gene Variants in Oxidative Stress Markers Between Mexican Patients with Type 2 Diabetes and ST-Segment Elevation Myocardial Infarction

**DOI:** 10.3390/antiox14080999

**Published:** 2025-08-15

**Authors:** Brenda Parra-Reyna, Iliannis Yisel Roa-Bruzón, Texali Candelaria García-Garduño, Luis Felix Duany-Almira, Antonio Quintero-Ramos, Jorge Ramón Padilla-Gutiérrez, Héctor Enrique Flores-Salinas, Emmanuel Valdes-Alvarado, José Francisco Muñoz-Valle, Yeminia Valle

**Affiliations:** 1 Institute for Biomedical Sciences Research, Department of Medical Clinics, University Center for Health Sciences, University of Guadalajara, Guadalajara 44340, Jalisco, Mexico; brenda.parra@academico.udg.mx (B.P.-R.); iliannis.roa8556@alumnos.udg.mx (I.Y.R.-B.); texali.garcia@alumnos.udg.mx (T.C.G.-G.); luis.duany8455@alumnos.udg.mx (L.F.D.-A.); ramon.padilla@academicos.udg.mx (J.R.P.-G.); emmanuel.valdes@academicos.udg.mx (E.V.-A.); jose.mvalle@academicos.udg.mx (J.F.M.-V.); 2 PhD Program in Human Genetics, Department of Molecular Biology, University Center for Health Sciences, University of Guadalajara, Guadalajara 44340, Jalisco, Mexico; 3 Immunology Laboratory, Department of Physiology, University Center for Health Sciences, University of Guadalajara, Guadalajara 44340, Jalisco, Mexico; antonio.qramos@academicos.udg.mx; 4 Cardiology Specialty, High Specialty Medical Unit, Western National Medical Center, Department of Cardiology, Mexican Social Security Institute, Guadalajara 44340, Jalisco, Mexico; hector.flores7136@academicos.udg.mx

**Keywords:** age, *CD36* gene, external factors, genetic susceptibility, MDA-LDL, OxLDL, sex, soluble CD36, Type 2 diabetes mellitus

## Abstract

Type 2 diabetes mellitus (T2DM) affects 90% of diabetes cases and worsens cardiovascular health by causing oxidative stress, which leads to oxidized LDL (oxLDL) and foam cell formation, contributing to atherosclerosis. This study examined the relationship between *CD36* gene variants, soluble CD36 (sCD36), oxLDL, and MDA-LDL in T2DM and ST-segment elevation myocardial infarction (STE-T2DM) patients in western Mexico. The analysis included 400 T2DM patients, 400 STE-T2DM patients, and 400 healthy controls. Results showed that STE-T2DM patients were older, mainly male, and had higher rates of smoking, sedentarism, and hypertension. Both diabetic groups exhibited elevated triacylglycerols and low HDL, with significantly higher C-reactive protein in STE-T2DM (*p* < 0.0001). No significant differences in *CD36* gene variant frequencies were found, but sCD36 levels were elevated in STE-T2DM, with associations to specific genotypes. oxLDL was higher in STE-T2DM compared to controls (*p* = 0.0268). Binary logistic regression analysis identified male sex, younger age, sedentarism, and rs3173798 T/T genotype as independent risk factors for myocardial infarction (AUC: 0.9267, *p* < 0.0001). Elevated sCD36 levels may reflect atherosclerosis progression in diabetes, indicating the need for further studies to clarify CD36’s role in cardiometabolic dysfunction. These findings highlight CD36’s involvement in oxidative stress responses through its interaction with oxLDL and MDA-LDL, suggesting its potential role as a molecular target in antioxidant defense mechanisms.

## 1. Introduction

Type 2 diabetes mellitus (T2DM) accounts for 90% of diabetes cases worldwide, and its prevalence is increasing globally [1]. T2DM is a significant comorbidity in myocardial infarction (MI) patients, present in 30% of hospitalized MI cases [2]. T2DM is associated with greater short- and long-term morbidity and mortality in patients with ST-segment elevation myocardial infarction (STEMI), who have a higher risk of bleeding, arrhythmias, heart failure, and death compared to non-T2DM patients [3].

Type 2 diabetes mellitus (T2DM) increases the risk of myocardial infarction through multiple mechanisms. Hyperglycemia and hyperinsulinemia impair endothelial function by reducing vasodilation and increasing oxidative stress [4]. T2DM also alters lipid metabolism by increasing small dense LDL particles that are prone to oxidation. These oxidized particles, including MDA-LDL and oxLDL, are taken up by macrophages via CD36, promoting foam cell formation and accelerating atherosclerosis [5,6]. These mechanisms contribute to the elevated cardiovascular morbidity and mortality observed in diabetic patients [7].

CD36 is an 88 kDa transmembrane glycoprotein involved in the uptake of oxLDL and regulation of lipid homeostasis. It plays a key role in various metabolic and cardiovascular disorders, including obesity, insulin resistance, dyslipidemia, and atherosclerosis [8,9]. Its expression is linked to insulin resistance due to increased fatty acid availability, and its role in foam cell generation is mediated by the uptake of MDA-LDL [5,10,11]. Elevated MDA-LDL levels have also been proposed as prognostic markers in coronary artery disease among individuals with T2DM [12,13].

The *CD36* gene, located on chromosome 7q11.2, comprises 15 exons. Single nucleotide variants (SNVs) such as rs3211938, rs3173798, and rs1761667 have been linked to T2DM and atherosclerosis by altering CD36 expression and function, potentially affecting lipid profiles and contributing to cardiovascular risk [9,14,15]. To date, these variants have not been studied in the Mexican population. Given that oxLDL and MDA-LDL are key products of lipid peroxidation during oxidative stress and that CD36 mediates their uptake in macrophages, this receptor plays a central role in redox imbalance and atherogenesis [8,16]. Therefore, understanding CD36’s genetic and soluble variants may provide insight into antioxidant pathways relevant to cardiometabolic diseases. Therefore, this study aimed to evaluate the association of these *CD36* SNVs with circulating levels of soluble CD36 (sCD36), MDA-LDL, and oxLDL in T2DM patients with and without STEMI, as well as in healthy individuals from western Mexico.

## 2. Materials and Methods

### 2.1. Study Population

The study included 800 clinically diagnosed patients; of these, 400 had ST-segment elevation myocardial infarction (STE-T2DM), and 400 had type 2 diabetes mellitus (T2DM) without a history of ischemic heart disease.

Inclusion Criteria:

For the cases, patients diagnosed with Type II Diabetes Mellitus (T2DM) according to the American Diabetes Association (ADA) were included, along with the co-occurrence of Acute Coronary Syndrome (ACS). This includes unstable angina or myocardial infarction (MI), with or without ST-segment elevation, classified based on the criteria set by the American College of Cardiology. These patients were selected from western Mexico.

Unstable angina refers to recent onset of angina pain or worsening of previously stable or prolonged angina at rest, with or without minimal electrocardiographic changes, and no increase in cardiac markers.

NSTEMI (Non-ST Elevation Myocardial Infarction) involves prolonged angina pain at rest, minimal electrocardiographic changes, no ST-segment elevation, or transient elevation, along with increased cardiac markers.

STEMI (ST-Elevation Myocardial Infarction) is characterized by prolonged angina pain at rest with characteristic electrocardiographic changes, including ST-segment elevation and a significant increase in cardiac markers.

The control group was based on a population of individuals without a history of ischemic heart disease, matched by age to the cases (over 45 years old) and recruited at the Mexican Institute of Social Security. These controls also had a diagnosis of Type II Diabetes Mellitus (T2DM) as per the ADA.

For all groups, participation was voluntary, and informed consent was obtained from each participant.

Non-Inclusion Criteria:

Individuals with underlying chronic heart diseases, such as myocarditis, pericarditis, hypertrophic cardiomyopathy, valvopathies, Tako–Tsubo cardiomyopathy, cardiac trauma, or congestive heart failure, were excluded. Additionally, those with non-cardiac diseases such as pulmonary embolism, pulmonary infarction, pneumothorax, pleuritis, pneumonia, anemia, aortic dissection, aortic aneurysm, esophageal spasm, or cerebrovascular disease were also excluded. Individuals with familial hypercholesterolemia, relatives of patients with hypercholesterolemia, or those who had received a blood transfusion within the last two months were also excluded.

For the control group based on the population, individuals with a personal history of ischemic heart disease, familial hypercholesterolemia, relatives, or those who had received a blood transfusion within the last two months were excluded.

Exclusion Criteria:

Exclusion was also applied to those who had incomplete molecular typing due to insufficient or poor-quality samples, lacked complete clinical history data, or requested to withdraw from the study.

### 2.2. Variables and Definitions for Study Participants

For the definition of clinical variables, the criteria were based on the 2019 ACC/AHA Guideline on the Primary Prevention of ASCVD [17], as described in Supplementary Table S6.

### 2.3. Ethical Approval and Informed Consent

Participants were recruited at the National Medical Center of the West, Jalisco, Mexico, and all subjects provided informed consent in accordance with the 2013 Declaration of Helsinki. This study was registered in the Research Committee (CI-CUCS), the Biosafety Committee (CBS-CUCS), and the Research Ethics Committee (CEI-CUCS) of the University Center of Health Sciences at the University of Guadalajara, under registration number CI-01614 (July 2019).

### 2.4. Biochemical Measurements

Venous blood samples were collected after overnight fasting. The biochemical parameters measured included glucose, triacylglycerols, total cholesterol, LDL cholesterol, HDL cholesterol, apolipoproteins A-I and B, and high-sensitivity C-reactive protein (hsCRP). Serum parameters were measured with standard kits of Biosystems (Costa Brava 30, Barcelona (Spain)) on an automated analyzer Mindray BS-120 (Reactivo Germania #9, Hermosillo, Sonora, México). In STE-T2DM patients, samples were collected approximately 48 h after the ischemic event, taking into account the patients’ pharmacological treatment, which included medications such as acetylsalicylic acid, clopidogrel, statins, heparin, enoxaparin, among others, all of which were considered in the statistical analysis.

### 2.5. Genotyping

Genomic DNA (gDNA) was extracted from whole venous blood using the modified Miller method, and its concentration was determined spectrophotometrically. Samples were stored at −20 °C until use. Genetic variants of the *CD36* gene (catalog numbers: C_8314999_10, C_12101686_10, and C_27468957_10 for rs1761667, rs3211938, and rs3179798, respectively) were genotyped using TaqMan Assays (Thermo Fisher Scientific Inc, Mexico City, México), following the manufacturer’s protocol. To ensure quality, double-blind genotyping was performed on one-fourth of the samples, with no variation in genotype assignment. Additionally, 15% of the samples were replicated to verify the genotypes.

### 2.6. Measurement of sCD36, oxLDL, and MDA-LDL

Plasma levels of soluble *CD36* (sCD36), oxidized low-density lipoproteins (oxLDL), and low-density lipoproteins modified by malondialdehyde (MDA-LDL) were measured in duplicate by ELISA assays using the human soluble CD36 kit from MyBioSource (MBS011248) (MyBioSource, Inc., San Diego, CA, USA) and human oxLDL and MDA-LDL kits (MBS265658 and MBS7254336, respectively) (MyBioSource, Inc., San Diego, CA, USA), following the manufacturer’s instructions. As a quality control, a coefficient of variation below 10% between replicates was accepted.

The term “oxidized LDL” refers to a variety of LDL preparations that have been oxidatively modified under defined ex vivo conditions. The main challenge when comparing results from different oxidized LDL studies is the heterogeneity of the preparations used. There is no widely accepted “gold standard” for preparing oxidized LDL ex vivo, and isolated tissue preparations can vary greatly between laboratories in terms of both composition and biological effects. Typically, oxidized LDL preparations are classified into two categories: “minimally modified” LDL (MM-LDL) and “oxidized” LDL (OxLDL). The main difference between them is that MM-LDL, although chemically distinct from unmodified LDL, is still recognized by the LDL receptor, while OxLDL is recognized by various scavenger receptors but not by the LDL receptor. The chemical and biological properties of oxidized LDL depend on the oxidizing agent and the oxidation conditions used, but most studies do not report the exact composition of LDLox or the oxidation conditions, making it difficult to compare their biological effects [18].

To obtain reference values, 400 healthy individuals (reference group, RG) were recruited, all with a normal glucose tolerance test and fasting glucose levels within the normal range. Additionally, they were ensured to have no personal pathological history of chronic diseases.

The RG was not intended for genetic comparisons. Instead of genotyping, a more accessible and feasible methodology was used, such as ELISA tests, which provides relevant information about biomarkers associated with type 2 diabetes and other metabolic diseases. This approach is particularly suitable given the high prevalence of type 2 diabetes and related metabolic issues in the Mexican population. Additionally, the costs associated with genetic testing are substantial and exceed the resources available for this study, which justified the choice of alternative methods like biochemical tests to analyze metabolic health indicators.

While these approaches are limited in genetic analysis, they are feasible within budgetary constraints and allow for meaningful research that contributes to understanding metabolic transitions and their link to chronic diseases in a high-risk population like the Mexican one.

In addition to the manufacturer’s instructions, the samples were analyzed in duplicate on each plate, and serial dilutions of the standard were included. It was verified that the coefficient of variation was below 10% for each replicate. Furthermore, efforts were made to ensure the representativeness of the three possible genotypes and clinical spectrum in the case of patients. Whenever possible, pairing was achieved based on sex, age, and the presence of cardiovascular risk factors.

### 2.7. Statistical Analysis

Statistical analysis was performed using R Studio 2024.09.1+394 (Bell Labs, Murray Hill, NJ, USA). The Mann–Whitney U test was used to analyze quantitative variables. Genetic variants were compared between groups using the chi-squared (χ^2^) test with a 95% confidence interval (CI). Associations were expressed as odds ratios (OR). The Hardy–Weinberg equilibrium was calculated from the genotype distribution. To estimate the relationship between cardiovascular risk factors (comorbidities, sex, medication use), binary and multivariable regression models were used. To control type I error due to multiple comparisons, Bonferroni correction was applied.

## 3. Results

A total of 800 patients with T2DM (400 with STEMI and 400 without) and 400 healthy controls were included. The patients with STE-T2DM were older, predominantly male, and had higher frequencies of smoking, sedentarism, and hypertension compared to the T2DM patients. The T2DM patients exhibited higher total cholesterol, triacylglycerols, LDL, Apo-AI, and Apo-B levels, while the CRP was significantly elevated in the STE-T2DM group. The HDL was low in both diabetic groups. (see Table 1 and Table 2).

As all participants had a previous clinical diagnosis of type 2 diabetes mellitus (T2DM), elevated glucose levels were expected in both groups. While the glucose levels in both groups were elevated and outside the normal range, the STE-T2DM group showed slightly higher values (median 187.5 mg/dL vs. 159 mg/dL, Table 2), though not statistically significant. This lack of significance may be due to the timing of sample collection in the STE-T2DM group, which occurred approximately 48 h after the ischemic event, during hospitalization and active pharmacological management. This likely mitigated the acute hyperglycemia typically seen in myocardial infarction. Therefore, the observed results might reflect post-infarction stabilization under standard care. In the T2DM group, the glucose levels were also elevated and exceeded the reference range. This finding is consistent with reports indicating that a substantial proportion of patients with type 2 diabetes mellitus do not achieve optimal glycemic control, despite being under medical treatment. Factors such as disease duration, treatment adherence, and presence of comorbidities may contribute to persistent hyperglycemia in this population [19]. In addition, HbA1c data were not included in the analysis due to inconsistent availability and the known limitations of interpreting HbA1c levels during acute cardiovascular events, which is acknowledged as a limitation of this study.

No significant differences were observed in the allelic or genotypic frequencies of the *CD36* SNVs (rs1761667, rs3173798, and rs3211938) between the STE-T2DM and T2DM groups. All variants conformed to the Hardy–Weinberg equilibrium. The rs3211938 G allele was rare in both groups (0.5% in STE-T2DM vs. 1.0% in T2DM), and no G/G homozygotes were detected. For rs3173798, the C allele frequencies were 13.1% in STE-T2DM and 14.1% in T2DM. For rs1761667, the G allele was present in 37.0% of STE-T2DM and 37.9% of T2DM cases. No significant differences were found under codominant, dominant, or recessive inheritance models (*p* > 0.25 for all comparisons). Haplotype analysis and linkage disequilibrium (LD) between rs1761667 and rs3173798 (D’ = 0.85) revealed no significant associations between groups. Full genotypic frequencies, odds ratios, and LD data are available in Supplementary Tables S1 and S2.

**Figure 1:**
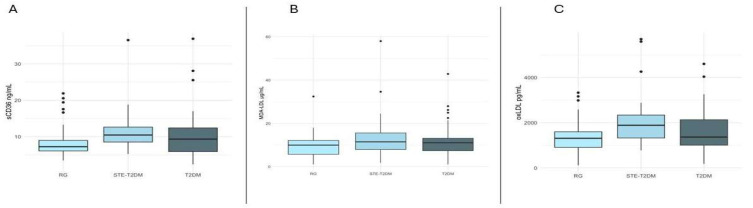
(**A**) sCD36 plasma levels among groups. Data are shown as median and interquartile range. RG: *n* = 80, 7.26 ng/mL (6.1–9.0 ng/mL); T2DM: *n* = 71, 9.38 ng/mL (5.83–12.42 ng/mL); STE-T2DM: *n* = 51, 10.66 ng/mL (8.36–12.65 ng/mL). RG vs. STE-T2DM: *p* < 0.0001; RG vs. T2DM: *p* = 0.0605; and STE-T2DM vs. T2DM, *p* = 0.0124. (**B**) MDA-LDL plasma levels in the study groups. Data are shown as median and interquartile range. RG: *n* = 46, 9.9 µg/mL (5.74–12.1 µg/mL), STE-T2DM: *n* = 47, 11.47 µg/mL (7.94–15.57 µg/mL), and T2DM: *n* = 42, 11.05 µg/mL (7.38–13.11 µg/mL). (**C**) oxLDL plasma levels in the study groups. Data are shown as median and interquartile range. RG: *n* = 23, 1312.5 pg/mL (910.2–1601.1 pg/mL); STE-T2DM: *n* = 31, 1888.6 pg/mL (1326.7–2341.7 pg/mL); T2DM: *n* = 23, 1367.3 (1009.0–2133.2 pg/mL). Comparisons were carried out through Kruskal–Wallis test, and *p*-values were obtained after Bonferroni post hoc test for correction.

The sCD36 levels were significantly higher in STE-T2DM patients (10.66 ng/mL) compared to T2DM (9.38 ng/mL) and controls (7.26 ng/mL, *p* < 0.0001). In STE-T2DM, the rs1761667 A/G genotype was associated with increased sCD36 levels (*p* = 0.0068). In T2DM, rs3173798 T/C and T/T genotypes were associated with reduced sCD36 levels (*p* < 0.0001). These associations remained significant after Bonferroni correction. A positive association was also observed between sCD36 and clinical factors such as reinfarction and hypertension, while overweight was associated with lower sCD36 (Figure 1A).

oxLDL levels were elevated in STE-T2DM compared to the reference group (1888.6 pg/mL vs. 1312.5 pg/mL, *p* = 0.0268). No other significant differences in oxLDL or MDA-LDL concentrations were detected across groups (Figure 1B,C). However, in T2DM patients, overweight and obesity were associated with higher MDA-LDL, and the rs3173798 T/C genotype was inversely associated with its levels. These associations did not remain significant after Bonferroni correction. Correlation analysis revealed a negative correlation between sCD36 and MDA-LDL in STE-T2DM (r = –0.30, *p* = 0.0402).

Binary logistic regression identified male sex, younger age, sedentary lifestyle, and rs3173798 T/T genotype as independent risk factors associated with STEMI in T2DM (AUC = 0.93, *p* < 0.01). The full regression outputs are available in Supplementary Tables S3–S5.

## 4. Discussion

### 4.1. Clinical and Biochemical Characteristics of the Study Population

This study analyzed three oxidative stress markers and their relationship with genetic variants located at their respective loci by comparing T2DM patients with and without a history of STEMI. We identified a high prevalence of cardiovascular risk factors—particularly smoking, sedentary lifestyle, metabolic syndrome, and hypertension—consistent with data from the Mexican population [20,21]. This clinical profile was reflected in biochemical alterations typical of T2DM, such as elevated ApoB and glucose levels. Interestingly, the T2DM group showed higher concentrations of total cholesterol, triacylglycerols, LDL-C, and ApoB than the STE-T2DM group, possibly due to intensive pharmacological management during hospitalization. This aligns with reports documenting post-infarction reductions of up to 39% in LDL-C, 47% in triacylglycerols, and 11% in HDL-C due to inflammation and lipid redistribution [22].

### 4.2. CD36 Gene Variants and Haplotype Distribution

Regarding the analyzed variants, the rs1761667 A>G polymorphism showed a distribution consistent with that reported by Ramos-López et al. [23] in western Mexican populations, with allele frequencies of 63% for A and 37% for G. The most frequent genotype was A/G, and no significant differences were observed between the STE-T2DM and T2DM groups under the dominant model (*p* = 0.4261).

Similarly, for rs3173798 T>C, the C allele frequency was 13.1% in STE-T2DM and 14.1% in T2DM, with low prevalence of the C/C genotype (~2%). This is consistent with its location in a conserved splice site of intron 3, which may limit its variability [24], and slightly lower than the 16% reported by Wojcik et al. (2019) in Mexican populations [25]. No inheritance model showed significant association with STEMI.

As for rs3211938 T>G, the G allele was rare (1% in STE-T2DM, 2% in T2DM), and no G/G homozygotes were detected. While Martín-Márquez et al. (2021) [26] reported complete absence of this allele in Mexican individuals, our findings are consistent with global reports that describe its near absence in non-African populations. Its limited frequency aligns with the estimated genetic ancestry in western Mexico, which includes approximately 9% African origin [27], but its rarity constrains its predictive value for *CD36* deficiency in this population. Significant linkage disequilibrium between rs3173798 and rs1761667 was identified in the reference group (D’ = 0.85, *p* < 0.05), indicating cohesive inheritance between these loci. This association may be due to their physical proximity within *CD36* or evolutionary pressures conserving certain allele combinations.

Haplotype analysis (rs3173798–rs1761667) revealed three common combinations—TA (~60%), TG (~23%), and CG (~17%)—with no significant differences between STE-T2DM and T2DM groups, suggesting haplotype stability despite linkage disequilibrium in the reference population. This supports the notion that *CD36*-related cardiometabolic risk is more likely driven by the independent effects of specific variants: rs3173798, associated with splicing and lipid metabolism [14,28], and rs1761667, known to influence transcriptional activity [14]. A similar lack of haplotype association with T2DM or dyslipidemia was reported in a Jordanian population [9], reinforcing that haplotypes may not underlie disease susceptibility. Nonetheless, gene–environment or gene–gene interactions—particularly with obesity, hypertension, or sedentary lifestyle—cannot be ruled out [11]. Larger, multilocus studies are needed to better understand the functional impact of *CD36* in cardiometabolic disorders.

### 4.3. Soluble CD36 as a Potential Biomarker

Plasma sCD36 levels were significantly higher in STE-T2DM patients compared to the T2DM and reference groups. Elevations of this marker have been linked to carotid intima-media thickening—a preclinical indicator of cardiovascular disease in both diabetic and non-diabetic individuals [16,29,30]. Nonetheless, its clinical role remains debated, as other studies have found no correlation between sCD36 and atherosclerosis imaging parameters [28].

From a molecular standpoint, hyperglycemia may activate PPAR-γ, promoting CD36 overexpression in macrophages and accelerating atherogenesis in diabetes [16,30]. CD36 is also implicated in lipid-induced insulin resistance and increased oxidized LDL uptake by monocytes, even in metabolically normal individuals. Additionally, its activation contributes to β-cell dysfunction and loss of pancreatic mass, thereby impairing insulin secretion [31].

Although no significant sCD36 differences were observed between the T2DM and reference groups—consistent with Castelblanco et al. (2017) [32]—other studies have reported elevated levels in diabetic subjects versus healthy controls [26,29] supporting its potential utility as a cardiovascular risk marker in specific populations.

### 4.4. Associations Between CD36 Variants and sCD36 Levels

No direct association was found between *CD36* polymorphisms (rs1761667, rs3173798, rs3211938) and STEMI risk in T2DM patients, nor with circulating sCD36 levels overall.

Regarding rs3173798, its C allele has been associated with lower HDL and increased cardiovascular risk [14]. Although no relationship was found with sCD36 levels in STE-T2DM, the T/T and T/C genotypes in T2DM were associated with reduced sCD36, suggesting a possible genotype-context regulatory effect. This aligns with evidence linking the variant to dyslipidemia, obesity, and diabetes—conditions closely tied to cardiovascular risk [14,33,34] It is important to note that several clinical variables, including sex, smoking status, hypertension, and sedentary lifestyle, differed significantly between the STE-T2DM and T2DM groups. These factors are known contributors to cardiovascular risk and could potentially confound the associations under study. To minimize bias, we incorporated these variables into the multivariable logistic and linear regression models. This adjustment allowed us to evaluate the independent effects of *CD36* gene variants and oxidative biomarkers on myocardial infarction risk. The significant associations observed—particularly for sCD36 levels and the rs3173798 T/T genotype—remained robust after controlling for these covariates.

### 4.5. Interpretation of Oxidative Biomarker Results

Regarding oxidative biomarkers, no significant MDA-LDL level differences were detected between groups. This contrasts with studies reporting elevated MDA-LDL in acute myocardial infarction patients (*p* < 0.001). Myocardial ischemia promotes reactive oxygen species generation and lipid peroxidation products like F2-isoprostanes, which enhance platelet aggregation and drive LDL oxidative modification in the vascular intima [35].

Statins use was associated with lower MDA-LDL levels, consistent with studies demonstrating these drugs’ oxidative stress-reducing effects [36]. A positive correlation between MDA-LDL and hypertension was also observed, aligning with Geshi et al. (2005) [37]. In T2DM, overweight and obesity were associated with higher MDA-LDL levels, while the rs3173798 T/C genotype showed an inverse association, suggesting genotype-dependent modulation influenced by body weight.

Regarding oxLDL, no significant group differences were found; however, in STE-T2DM, its concentration was negatively associated with amlodipine use. Previous studies have demonstrated that this drug reduces LDL oxidizability and enhances antioxidant defenses via the glutathione system and nitric oxide synthase activity, which may explain the observed trend [38].

These findings support the protective effect of certain treatments on oxidative stress biomarkers. Nevertheless, a larger cohort and stratified analysis are required to validate these associations and deepen the understanding of pharmacological treatment, genetic profile, and oxidative stress interactions in T2DM patients.

### 4.6. Study Limitations and Future Directions

Although no significant associations were identified between *CD36* polymorphisms (rs1761667, rs3173798, rs3211938) and plasma levels of sCD36, MDA-LDL, or oxLDL, these results do not rule out a functional CDS6 role in oxidative stress and cardiovascular risk in T2DM. Variants not included here, such as rs1527483, or extended haplotype combinations, may influence biomarker variability. Indeed, rs3211938 has been previously linked to glucose, oxLDL, and insulin in Mexican T2DM patients. Moreover, linkage disequilibrium between rs3173798 and rs1761667 observed in the reference group suggests functional haplotypic configurations that our study was underpowered to detect due to sample size or low allele frequencies.

*CD36* expression and its soluble form may be modulated by epistatic interactions, clinical conditions (hyperglycemia, obesity, hypertension), and environmental factors like sedentary lifestyle or smoking. Post-transcriptional mechanisms—including microRNA regulation and hyperglycemia-induced mitochondrial dysfunction—also play a role. These complex genetic, metabolic, and environmental interactions may explain the absence of detectable associations, particularly in highly heterogeneous mestizo populations such as western Mexico.

Future studies should include broader *CD36* gene analysis, extended haplotype exploration, gene–gene interaction investigation, and multiomic or epigenetic approaches to elucidate mechanisms linking *CD36* with metabolic dysfunction and atherogenesis in T2DM.

## 5. Conclusions

In conclusion, our study highlights the complex interplay between *CD36* gene variants, circulating oxidative biomarkers, and cardiovascular risk in Mexican patients with T2DM. While no direct associations were observed between the analyzed polymorphisms and the presence of STEMI, elevated sCD36 levels in STE-T2DM patients suggest its potential role as a marker of cardiovascular stress or endothelial activation. Additionally, the rs3173798 T/T genotype was associated with lower sCD36 levels in T2DM patients, pointing to possible genotype-context regulatory mechanisms.

Although MDA-LDL and oxLDL did not differ significantly between groups, trends observed in relation to pharmacological treatments underscore the importance of medication in modulating oxidative stress. These findings support the relevance of considering clinical context and comorbidities when interpreting biomarker data.

Future studies should explore extended haplotypes, gene–gene and gene–environment interactions, and multiomic approaches to better understand the contribution of *CD36* to oxidative stress and atherogenesis. Such insights may pave the way for more personalized prevention strategies in high-risk diabetic populations.

## Figures and Tables

**Table 1:** Demographic and clinical characteristics of the studied groups.

Variables	STE-T2DM (*n* = 400) *n* (%)	T2DM (*n* = 400) *n* (%)	*p*
*Age, years*	63 (57–66)	58 (52–64)	ns
*Male* *Female*	329 (82.25) 71 (17.75)	194 (48.50) 206 (51.50)	0.0001
*Sedentarism*	260 (65.00)	92 (23.00)	0.0001
*MeS*	260 (65.00)	272 (68.00)	ns
*Dyslipidemia*	228 (57.00)	116 (29.00)	0.002
*Smoking*	301 (75.25)	153 (38.25)	0.001
*Overweight*	172 (43.00)	37 (9.25)	ns
*Obesity*	100 (25.00)	31 (7.75)	ns
*Hypertension*	200 (50.00)	87 (21.75)	0.0001

Data are expressed as median (interquartile range) for continuous variables and frequency (percentage) for categorical variables. Diabetic patients with ST-segment elevation myocardial infarction: STE-T2DM, and diabetic patients: T2DM. Fisher’s exact test was used due to expected frequencies. HBP: high blood pressure; MeS: metabolic syndrome; ns: not significant.

**Table 2:** Paraclinical characteristics of the studied groups.

	STE-T2DM	T2DM	Reference Values	*p*
*Cholesterol*	108.5 (77–126)	156 (138–185)	150–199 (mg/dL)	<0.0001
*Glucose*	187.5 (162–211)	159 (118–244)	75–105 (mg/dL)	ns
*Triacylglycerols*	84 (76–93)	114 (84–147)	≤250 (mg/dL)	0.00056
*LDL*	39 (28–52)	68 (52–95)	<130 (mg/dL)	<0.0001
*HDL*	18 (12–24)	34 (25–51)	>40 (mg/dL)	<0.0001
*CRP*	24 (8.4–33.7)	3 (1–4)	1–10 (mg/dL)	<0.0001
*Apo-AI*	156 (138–170)	194 (173–210)	94–178 (mg/dL)	<0.0001
*Apo-B*	121 (101–152)	163 (148–184)	63–133 (mg/dL)	<0.001

STE-T2DM: patients with type 2 diabetes mellitus and ST-segment elevation myocardial infarction; T2DM: patients with type 2 diabetes mellitus; LDL: low-density lipoprotein; HDL: high-density lipoprotein; CRP: C-reactive protein; Apo-AI: apolipoprotein-AI; Apo-B: apolipoprotein-B. The Mann–Whitney U test was performed. The values were established according to the AMORIS and INTERHEART studies. Data were expressed as median and interquartile range. TnI: 6.73 (2.04–21.65). ns: not significant.

## Data Availability

Data is contained within the article and Supplementary Materials.

## Supplementary Materials

The following supporting information can be downloaded at https://www.mdpi.com/article/10.3390/antiox14080999/s1. Table S1. Genotypic and allelic frequencies of the study groups. Table S2. *CD36* Gene Haplotypes. Table S3. Linear Regression Analysis of Factors Associated with Plasma sCD36 Levels in the STE-T2DM Group. Table S4. sCD36 and MDA-LDL linear regression analysis in T2DM group. Table S5. Binary logistic regression. Table S6. Variables and Definitions for Study Participants.

## Abbreviations

The following abbreviations are used in this manuscript:

STEMI	ST elevation myocardial infarction
NSTEMI	Non-ST elevation myocardial infarction
