# Supplementary material for: Implications of CD36 Gene Variants in Oxidative Stress Markers Between Mexican Patients with Type 2 Diabetes and ST-Segment Elevation Myocardial Infarction
Source: Antioxidants (Basel). 2025 Aug 15;14(8):999. doi: 10.3390/antiox14080999 (PMC12383103; doi:10.3390/antiox14080999)
Supplement: Supplementary file 1 [file antioxidants-14-00999-s001.zip › antioxidants-3778984-supplementary.pdf]

Supplementary Materials

Supplementary material related to "Implications of CD36 Gene Variants in Oxidative Stress Markers Between Mexican Patients with Type 2 Diabetes and ST-segment elevation myocardial infarction"

Supplementary Table S1. Genotypic and allelic frequencies of the study groups.

	CD36 Allele/Genotype	STE-T2DM n (%)	T2DM n (%)	OR (CI 95%)	*p*
rs3211938 T>G		**n=400**	**n=400**		
Allele	T	796 (99.5)	792 (99.0)		
	G	4 (0.5)	8 (1.0)	0.4975 (0.1492-1.6588)	0.25
Genotype	T/T	396 (99.0)	392 (98.0)	-	-
	T/G	4 (1.0)	8 (2.0)	1.9798 [0.5913-6.6283]	0.26
	G/G	0 (0)	0 (0)	0.9899 [0.0196-50.0160]	0.99
Dominant model	T/G + G/G	4 (1.0)	8 (2.0)	0.4949 [0.1478-1.6571]	0.25
Recessive model	T/T + T/G	400 (100.0)	400 (100.0)	1.00 [0.01-50.52]	1.00
rs3173798 T>C		**n=400**	**n=400**		
Allele	T	695 (86.9)	687 (85.9)		
	C	105 (13.1)	113 (14.1)	0.9091 (0.6835-1.2093)	0.5128
Genotype	T/T	303 (75.7)	293 (73.2)	-	-
	T/C	89 (22.3)	101 (25.3)	0.8521 (0.6144-1.1818)	0.3376
	C/C	8 (2.0)	6 (1.5)	1.2893 (0.4420-3.7613)	0.6418
Dominant model	T/C + C/C	97 (23.8)	107 (26.7)	0.8766 (0.6377-1.2051)	0.4174
Recessive model	T/T + T/C	392 (98.0)	394 (98.5)	0.7462 (0.2565-2.1705)	0.5910

rs1761667 G>A		N=400	N= 400	OR	p
Allele	A	504 (63.0)	497 (62.1)		
	G	296 (37.0)	303 (37.9)	0.9633 (0.7867-1.1796)	0.7177
Genotype	A/A	163 (40.8)	152 (38.0)	-	-
	A/G	178 (44.5)	193 (48.2)	0.8600 (0.6368-1.1616)	0.3255

	G/G	59 (14.7)	55 (13.8)	1.0003 (0.6516-1.5358)	0.9988
Dominant model	A/G + G/G	237 (59.3)	248 (62.0)	0.8912 (0.6710-1.1836)	0.4261
Recessive model	A/A+A/G	341 (85.2)	345 (86.2)	0.9214 (0.6197-1.3700)	0.6858

*Chi-square; CI: confidence interval; OR: odds ratio. STE-T2DM: patients with ST-elevation myocardial infarction and type 2 diabetes. T2DM: patients with type 2 diabetes but no history of infarction. Comparisons were made to evaluate genetic associations. All variants were in Hardy-Weinberg equilibrium. No significant differences were found between groups under codominant, dominant, or recessive models.

Supplementary Table S2. CD36 Gene Haplotypes

Haplotypes	STE-T2DM (n=400) n (%)	T2DM (n=400) n (%)	*OR (95% CI)*	*p*
TA$^{\varphi}$	252 (63.00)	240 (60.00)	1	-
TG	104 (26.00)	92 (23.00)	1.014 (0.683 – 1.506)	0.943
CG	44 (11.00)	68 (17.00)	0.885 (0.521 – 1.503)	0.652

Odds ratio (OR), 95% confidence interval (CI). Haplotypes with a frequency lower than 3% were excluded from the analysis. Diabetic patients with myocardial infarction: STE-T2DM; Type 2 diabetic patients: T2DM.

Linkage disequilibrium was observed between rs3173798 and rs1761667 in the RG group (D' = 0.85, $p < 0.05$). No significant differences in haplotype frequencies were found between groups, excluding haplotypes with frequency <3%.

Supplementary Table S3. Linear Regression Analysis of Factors Associated with Plasma sCD36 Levels in the STE-T2DM Group

**Cardiovascular risk factors**	
**Independent variable**	**β coefficient, (95% CI), p-value**
Reinfarction	6.61 (3.35, 9.86), *0.0047*
Normal weight	4.57 (1.48, 7.67), 0.7595
Overweight	-4.44 (-7.70, -1.18), *0.0087*
Dyslipidemia	3.16 (0.65, 5.66), *0.0145*

**Pharmacological treatment and genetic variants**	
**Independent variable**	**β coefficient, (95% CI), p-value**
β-blockers	2.19 (-0.50, 4.89), 0.1085
Renin-angiotensin inhibitor	-2.87 (-5.83, 0.08), 0.0563
Diuretics	2.55 (-0.25, 5.36), 0.0732
rs1761667 A/G	4.03 (1.17, 6.89), *0.0068*

High blood pressure	3.65 (0.97, 6.332), *0.0086*
Smoking	2.89 (0.46, 5.32), 0.2060
Sedentarism	-1.29 (-3.78, 1.20), 0.3027

rs1761667 G/G	3.26 (-0.17, 6.69), T0.0623

Cardiovascular risk factors adjusted R^2: 0.31, model p-value: 0.0016. Pharmacological treatment adjusted R^2: 0.14, model p-value: 0.0382. Although the amount of variability explained in this model is not relatively high, the relationship between the variables is statistically significant.

Regression coefficients (β), 95% confidence intervals (CI), and p-values are shown for clinical variables, pharmacological treatments, and CD36 genotypes. Significant associations after Bonferroni correction are indicated in bold.

Supplementary Table S4. sCD36 and MDA-LDL linear regression analysis in T2DM group.

**MDA-LDL plasma levels**	
**Independent variable**	**β coefficient, (95% CI), p-value**
rs3173798 T/C	-64.64, (-120.64, -8.64), *0.0249*
rs3173798 T/T	-54.88, (-109.84, 0.082), 0.0503
Dyslipidemia	-15.44, (-33.01, 2.122), 0.0830
Smoking	-17.34, (-38.24, 3.55), 0.1010
Overweight	22.60, (2.49, 42.71), *0.0286*
Obesity	24.99, (1.36, 48,61), *0.0387*

**sCD36 plasma levels**	
**Independent variable**	**β coefficient, (95% CI), p-value**
rs3173798 T/C	-33.06 (-47.20, -18.92*),* *<0.0001*
rs3173798 T/T	-31.89 (-45.76, -18.02) , *<0.0001*
Smoking	-2.65 (-7.41, 2.11), 0.2701
Sedentarism	5.19 (1.06, 9.32), *0.0146*

Linear regression model for MDA-LDL: adjusted R^2: 0.17, model p-value: 0.0483. Linear regression model for sCD36: adjusted R^2: 0.25, model p-value: 0.0001.

Multivariable linear regression analysis of plasma sCD36 levels in STE-T2DM and T2DM groups. Variables include cardiovascular risk factors, pharmacological treatment, and CD36 genotypes. Bonferroni correction was applied separately for each group of predictors. In the STE-T2DM group, only the rs1761667 A/G genotype remained significantly associated with sCD36 levels after correction (adjusted p = 0.034). In the T2DM group, rs3173798 T/C and T/T genotypes were significantly associated with lower sCD36 levels ($p < 0.0001$), while sedentary lifestyle showed a borderline effect. Full regression coefficients and p-values are shown.

Supplementary Table S5. Binary logistic regression.

**Variable**	**OR (95% CI), p-value**
Age	0.85, (0.70, 0.96), *0.0362*
Sex	4.56 (1.90, 8.46), *0.0046*
rs1761667 A/G	1.16 (-1.3, 4.04), 0.3804
rs1761667 G/G	3.99 (0.53, 8.85), 0.0510
rs3173798 T/T	7.97 (3.43, 14.99), *0.0055*
MDA-LDL	0.95 (0.83, 1.06), 0.4326
oxLDL	0.99 (0.99, 1.08), 0.7464
Dyslipidemia	-2.19 (-5.46, 0.23), 0.1134
Sedentarism	0.03 (0.001, 0.29), *0.0083*

AIC: 51.013, AUC: 0.9267, McFadden R^2: 0.8117, p value of global model: <0.0001, where a Likelihood Ratio and chi-square test.

Multivariate linear regression analyses were conducted to evaluate the associations of age, sCD36, MDA-LDL, and oxLDL levels in the reference group (RG). Age was negatively associated with sCD36 and positively associated with MDA-LDL. sCD36 levels showed a stronger correlation with MDA-LDL than with oxLDL. In the STE-T2DM group, a negative correlation was observed between sCD36 and MDA-LDL (r = –0.30, p = 0.0402), while in the T2DM group, a positive correlation was found between oxLDL and MDA-LDL (r = 0.43, p = 0.0485). No significant correlations were found between oxLDL and sCD36 in any group. Logistic regression identified age, male sex, sedentary lifestyle, and the rs3173798 T/T genotype as independent predictors of STEMI in T2DM patients. Complete model outputs are provided.

Supplementary Table S6. Variables and Definitions for Study Participants

Variables	
Age, years	According to their chronological age, understood as the number of completed years from birth to the time of data collection. In this study, all participants belong to the adult population, defined as individuals aged 18 years or older.
Biological sex	According to the physiological, anatomical, and genetic characteristics that distinguish individuals as male or female, determined at the time of birth.
Sedentarism	Individuals who spend more than 60% of their daily waking hours sitting, reclining, or lying down, engaging in activities with low or no energy expenditure, such as watching television or using electronic devices, exhibit sedentary behavior. This includes spending more than 9 hours per day sitting (considering a 15-hour waking day), with an energy expenditure of 1.5

	METs or less, which will be measured through self-reports or screen time records. Additionally, sedentary behavior is defined if the time spent in front of screens (without associated physical activity) exceeds 2 hours per day and if the individual does not meet the minimum physical activity recommendations of the WHO (150 minutes of moderate activity per week).
MeS	Adults diagnosed with metabolic syndrome, which is defined by the presence of three or more metabolic abnormalities. These include a waist circumference greater than 40 inches (102 cm) in men and 35 inches (88 cm) in women, a serum triglyceride level of 150 mg/dL or higher; high-density lipoprotein (HDL) cholesterol less than 40 mg/dL in men or less than 50 mg/dL in women, or treated; a fasting glucose of 100 mg/dL or higher; and a systolic blood pressure of 130 mm Hg or higher or a diastolic blood pressure of 85 mm Hg or higher, or treated.
Dyslipidemia	Adults diagnosed with dyslipidemia, defined by abnormal lipid levels in the blood. Participants must meet at least one of the following criteria: LDL cholesterol greater than 190 mg/dL; HDL cholesterol less than 40 mg/dL in men or 50 mg/dL in women, triglycerides greater than 150 mg/dL, or total cholesterol greater than 240 mg/dL. The evaluation was conducted through a lipid profile, measuring total cholesterol, LDL, HDL, and triglyceride levels.
Smoking	Any individual who has smoked or is currently smoking cigarettes daily for any amount over the past month.
Overweight	Adults with a Body Mass Index (BMI) between 25 kg/m^2 and 29.9 kg/m^2.
Obesity	Adults with a BMI greater than or equal to 30 kg/m^2, classified into the following categories: Class I (BMI from 30 to 34.9 kg/m^2), Class II (BMI from 35 to 39.9 kg/m^2), and Class III (BMI greater than 40 kg/m^2).
Hypertension	Patients with a prior diagnosis of Hypertension based on a clinical blood pressure of systolic blood pressure greater than 130 mmHg or diastolic blood pressure greater than 80 mmHg. The diagnosis is confirmed through repeated blood pressure measurements in a clinical setting or through ambulatory blood pressure

	monitoring (ABPM) or home blood pressure monitoring (HBPM).
